# Prevalence of Overweight and Obesity in 6–7-Year-Old Children—A Result of 9-Year Analysis of Big City Population in Poland

**DOI:** 10.3390/ijerph17103480

**Published:** 2020-05-16

**Authors:** Joanna Szczyrska, Agnieszka Jankowska, Michał Brzeziński, Marek Jankowski, Paulina Metelska, Agnieszka Szlagatys-Sidorkiewicz

**Affiliations:** 1 Department of Pediatrics, Gastroenterology, Allergology and Nutrition, Faculty of Medicine, Medical University of Gdańsk, 80-803 Gdańsk, Poland; ajankowska@gumed.edu.pl (A.J.); brzezinski@gumed.edu.pl (M.B.); agnieszka.szlagatys-sidorkiewicz@gumed.edu.pl (A.S.-S.); 2 Gdańsk Center for Health Promotion, 80-397 Gdańsk, Poland; marek.jankowski@opz.gdansk.pl; 3 Department of Public Health and Social Medicine, Faculty of Health Sciences, Medical University of Gdańsk, 80-210 Gdańsk, Poland; metelska.paulina@gumed.edu.pl

**Keywords:** prevalence, obesity, overweight, child, Poland

## Abstract

Excess body weight is a serious public health problem, which affects both adults and children. Unfortunately, the prevalence of excess body weight in children in Poland is not subject to any regular screening. The aim of this study was to analyze the prevalence of overweight and obesity in 6–7-year-old children in the municipality of Gdańsk in the years 2008–2016. The anthropometric parameters of 12,330 children (49.55% girls and 50.45% boys) with a mean age of 6.53 ± 0.38 years were analyzed. The prevalence of overweight was 7.49% (7.91% girls and 7.07% boys) and obesity 4.24% (4.47% girls and 3.99% of boys). There was no difference in the prevalence of neither overweight nor obesity between boys and girls (*p* = 0.076). However, the prevalence of overweight and obesity combined is higher in girls (12.38% vs. 11.06%, *p* = 0.023). There were no statistically significant differences in the prevalence of overweight and obesity neither in the group of girls nor in the group of boys in children aged 6–7 years in yearly cohorts examined between 2008 and 2016. The prevalence of excess body weight in this population is at a stable level.

## 1. Introduction

Excess body weight is a serious public health issue both in Poland and worldwide. It affects not only adults but also children. Itself a disease, obesity also significantly increases the risk of developing many diseases of affluence. The occurrence of overweight and obesity during childhood is associated with a clearly higher risk of obesity and its consequences in adulthood [1,2].

The number of children with overweight and obesity is still alarmingly high despite the slowdown in the previously soaring trend, which is still observed in most developed countries [3,4]. In developing countries, the numbers of obese and overweight children are still growing.

According to WHO, 41 million children worldwide under the age of 5 were overweight or obese; moreover, over 340 million children and adolescents aged 5–19 were overweight (18% girls, 19% boys) or obese (6% girls, 8% boys) in 2016 [5]. As soon as possible during childhood, preventive and curative measures should be undertaken, and it is crucial to know the scale of the problem in order to plan them correctly. Unfortunately, the prevalence of excess body weight in children in Poland is not a subject to any regular screening. Accurate data is not available. Moreover, the epidemiological studies published so far are based on different age groups and different criteria for diagnosing overweight and obesity in children, which makes any comparative analysis difficult.

The aim of this study was to analyze the occurrence of excess body weight in 6–7-year old children in the municipality of Gdansk in 2008–2016, based on published data we hypothesize that there is no significant increase.

## 2. Materials and Methods

The anthropometric parameters of 6–7-year-old children living in Gdańsk collected as part of the screening test conducted in this age group by the Gdańsk Children’s Promotion Center (currently Gdansk Center for Health Promotion) “Healthy Life of Your Child” were analyzed. The program is financed by the City of Gdańsk, and its main goal is to promote a healthy lifestyle, assess health and psychophysical development as well as to identify the risks to the child’s health. The study comprised 12,330 children (49.55% girls and 50.45% boys) with a mean age of 6.53 ± 0.38 years, participating in the Your Child’s Healthy Life program in the period from December 2007 to August 2016 (Table 1). These children were born in years 2001–2009 and comprise about one third of children born in Gdańsk in that period.

Anthropometric measurements of all children were taken. Body height was measured to the nearest 1 mm, with the children standing in the Frankfurt position, barefoot. Body weight was assessed to the nearest 100 g, with the children barefoot and dressed in their underwear or gym outfits. On the basis of the height and weight measurements, the Body Mass Index (BMI) was calculated according to the applicable formula. The BMI value was referenced to percentile grids developed as a part of the OLAF and OLA projects [6,7]. In accordance with the criteria for diagnosing excess body weight in Poland, the BMI ≥ 85 percentile was assumed as overweight and BMI ≥ 95 as obesity.

Normal distribution of continuous variables was verified with the Shapiro–Wilk test. Descriptive statistics are presented as the mean or median and standard deviation from the mean. Chi-squared and Kruskal–Wallis’ tests were used. All statistical tests were 2-tailed and performed at the 0.05 significance level. Statistical analyses were performed with the Statistica 13 (TIBCO Software Inc., Tulsa, OK, USA 2017).

### Ethics Approval

The approval for this study was approved by Independent Bioethics Committee for Scientific Research of Medical University of Gdansk (decision no. NKBBN/228/2012 and NKBBN/228-197/2014).

## 3. Results

The results of the mean body weight, height, BMI and BMI percentile for the entire studied population as well as by sex and year of birth are presented in Table 1, Table 2 and Table 3. In the examined group, boys had a higher mean body weight (23.66 ± 4.24 vs. 23.08 ± 4.14), body height (122.35 ± 5.52 vs. 121.33 ± 5.53) and BMI (15.72 ± 1.9 vs. 15.56 ± 1.9), while girls had higher mean BMI percentile (47.97 ± 27.42 vs. 45.21 ± 27.03) (*p* < 0.000).

Analyzing the mean BMI value and mean BMI percentile by year of birth, significant differences were found between girls born in 2007 and those born in 2008 (*p* = 0.029 and *p* = 0.031, respectively). Among boys, higher mean BMI and mean BMI percentile were found in boys born in 2002 compared to boys born in 2006 (*p* = 0.021 and *p* = 0.018).

**Figure 1:**
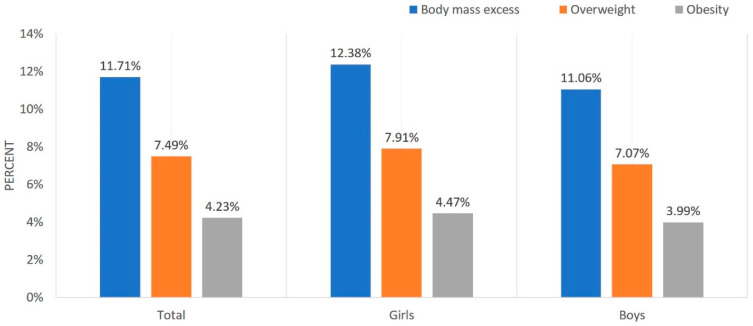
Prevalence of overweight and obesity in the study group.

The prevalence of overweight in the studied population was 7.49% (7.91% in girls and 7.07% in boys) and obesity (BMI ≥ 95^th^ percentile), 4.24% (4.47% in girls and 3.99% in boys)—Figure 1. There was no difference in the prevalence of either overweight or obesity between boys and girls (*p* = 0.076). However, excess body weight, defined as either overweight or obesity, occurred more often in girls than in boys (12.38% vs. 11.06%, *p* = 0.023).

**Figure 2:**
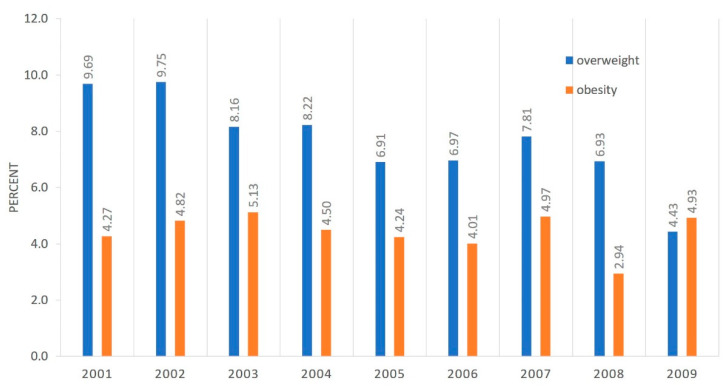
Prevalence of overweight and obesity at age 6-7 in girls by birth year.

**Figure 3:**
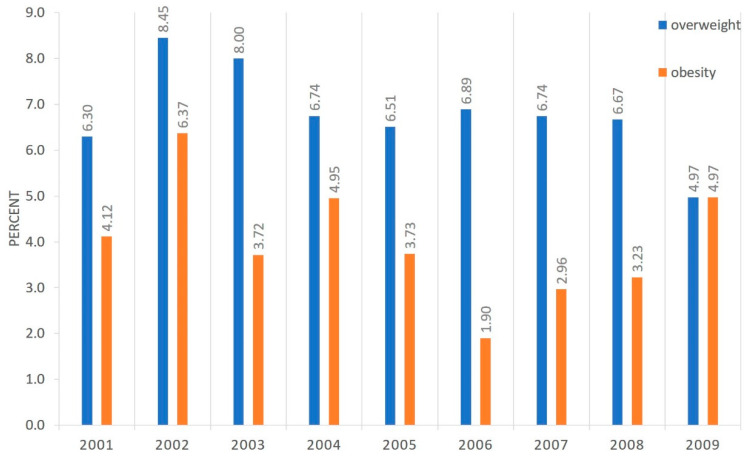
Prevalence of overweight and obesity in boys at age 6–7 by birth year.

**Figure 4:**
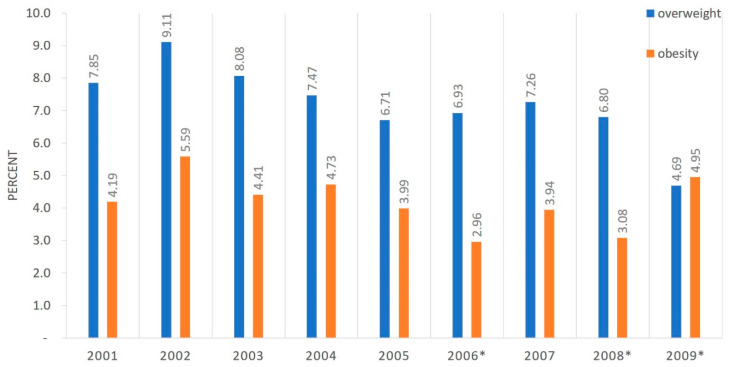
Prevalence of overweight and obesity in all children (boys and girls) at age 6–7 by birth year. (* 2006–2008: *p* = 0.0043; 2006–2009: *p* = 0.0026; 2008–2009: *p* = 0.0053).

There were no statistically significant differences in the prevalence of overweight and obesity in children aged 6–7 years neither in the group of girls nor in the group of boys in yearly cohorts examined between 2008 and 2016 (born 2001–2009)—Figure 2 and Figure 3. The prevalence of overweight and obesity in all children by birth year can be found in Figure 4. Differences were found between some birthyears but with no clinical or populational importance and no trend was observed. Analyzing excess body weight (overweight and obesity combined), no differences were found in its prevalence when considering birth years in neither girls, nor boys.

## 4. Discussion

Summing up the results presented above, in the studied group of children born in the years 2001–2009, a stable prevalence of excess body weight at the age of 6–7 years old was observed.

In order to relate the obtained results to the available data, one must take into account certain limitations resulting from the fact that the epidemiological studies published so far concerning the prevalence of excess body weight in children in Poland, Europe and worldwide are based on different age groups and different criteria for recognizing overweight and obesity in children (e.g., WHO, IOTF, national criteria) [8,9]. In our study, the current Polish criteria based on actual reference charts for Polish children (OLAF percentile grids) were used.

The representative sample of Polish children (on which basis the OLAF percentile girds were prepared) was analyzed by Kułaga et al. using three different international diagnostic criteria for child overweight and obesity (IOTF, CDC, WHO) [10]. Depending on criteria used, overweight (including obesity) in 7-year-old children was observed in 17.6% (IOTF), 19.4% (CDC) or 21.7% (WHO) of girls and 19.5% (IOTF), 22.6% (CDC) or 27.6% (WHO) of boys. The prevalence of obesity in girls was assessed at 4.6% (IOTF), 7.1% (CDC) or 5.9% (WHO) and in boys at 5.3% (IOTF), 11.2% (CDC) or 11.4% (WHO). The observed differences in the prevalence of excess body weight result from different BMI values set as cut-off points depending on the criteria used. In the case of the Polish criteria (85th and 95th percentile, OLAF percentile grids) the BMI cut-off points for 7-year-old children are BMI values of 17.9 and 20.0 kg/m^2^ in girls, 18.2 and 20.4 kg/m^2^ in boys respectively for overweight and obesity. Regardless of sex, these are higher than the WHO’s BMI cut-off values—17.3 and 19.8 kg/m^2^ in girls, 17.0 and 19.0 kg/m^2^ in boys. Therefore, the estimated prevalence of excess body weight in 7-year-old children based on the Polish criteria will be lower than when using the WHO criteria, but this difference will be smaller in the case of obesity in girls.

In the published research on the Polish population, the closest age group to ours was examined in a nationwide study conducted in 2001 by Małecka-Tendera et al. on a representative group of children aged 7–9 years old [11]. In diagnosing overweight and obesity, IOTF criteria were used. Overweight and obesity were found in 15.4% of children (15.8% girls and 15% boys), including obesity in 3.6% (3.7% girls and 3.6% boys). This study also compared the prevalence of excess body weight in children in Poland and in France. The prevalence of obesity in Polish children was comparable to the French children (3.6% vs. 3.7%), and no significant difference in overweight prevalence in girls was found (15.8% in Poland vs. 18.3% in France). In boys, however, a slight difference was noted (15.0% in Poland vs. 17.9% in France), with a significantly higher frequency of overweight reported in 9-year-old French boys. Moreover, both overweight and obesity declined with age in Polish children, which was not observed in the French study.

According to the European research project COSI (Childhood Obesity Surveillance Initiative), excess weight affects almost one-third of Polish 8-year-olds (30.7%, *n* = 1047) [12]. Overweight and obesity are more common in boys (32.4%, *n* = 552) than in girls (29.1%, *n* = 495). The COSI study uses WHO 2007 recommendations to define overweight and obesity. However, using the criteria recommended by the IOTF, excess weight was found in 23.2% of children aged 8 years, and the occurrence of overweight and obesity was not sex-dependent (*p* = 0.467). A similar percentage of children with excess body weight was observed when using the OLAF national standards (22.8%, *N* = 776), but in this case overweight and obesity were statistically significantly more common in girls (24.4%, *n* = 414, *p* < 0.05). This example perfectly illustrates the problem with using different criteria—depending on the cut-off point, the prevalence of excess body weight varied by almost 8% in the same population, while gender effects coved the entire spectrum—boys’ advantage, girls’ advantage and no differences due to sex. It was the fourth round of the COSI study (2015–2016) and the first in which the study was conducted on the population of Polish children. The Polish COSI sample included a randomly selected group of 3408 children aged 8 years from the area of 9 voivodeships.

So far, for over 10 years of the COSI project, a significant decrease in the prevalence of both overweight and obesity in children aged 6–9 years in Greece, Italy, Portugal and Slovenia was observed. A decreasing tendency was also observed in Ireland and Spain. Belgium, Czech Republic and Norway had stable prevalence. An increasing tendency in obesity was observed among Latvian girls, as well as Bulgarian and Lithuanian boys [13].

One of the latest systematic reviews and meta-analyses published in JAMA Pediatrics in August 2019 concerns the prevalence of overweight and obesity in European children in the years 1999–2016 [4]. It covers 103 studies from 28 countries. Children aged 2–13 years old showed stabilization in the prevalence of excess body weight in most European countries, but despite this stabilization, the prevalence is still very high. The growing prevalence in the Mediterranean countries was considered particularly worrying.

As for worldwide trends in BMI, overweight, and obesity, the NCD Risk Factor Collaboration presented a metanalysis of 2416 population-based measurement studies in 128.9 million children, adolescents and adults in years 1975–2016 [3]. Their main conclusion was that the children’s BMI rising trends have plateaued around the year 2000 in high-income countries (they remain at high levels though). On the other hand, they have accelerated in some parts of Asia and are no longer correlated with those of adults. Regional changes in eastern Europe in age-standardized mean BMI charts were found insignificant for both boys and girls born between 1975 and 2016.

We did not find any long-term Polish nationwide studies on the prevalence of overweight and obesity in children. Only a few regional studies from different regions of Poland can be found [14,15,16,17,18]. In a study on the prevalence of overweight and obesity in school children from south-eastern Poland between 1998 and 2008, Mazur et al. (who used IOTF criteria) reported a trend toward stabilization of the prevalence of overweight and obesity. The declining prevalence of obesity in girls (10.1% in 1998 vs. 7.7% in 2008) and increased prevalence of overweight in boys (10.5% in 1998 vs. 14.2% in 2008) might suggest that in this age group of children, the secular trend is gender dependent [16].

Brzeziński et al. analyzed the prevalence of overweight and obesity in children in the city of Gdańsk in the years 1992–2012, covering 70,329 children aged 6–13 years [17]. There was no consistent trend of increasing or decreasing prevalence of excess body weight in any age group of studied children. However, in the group of 6–7-year-old children born in the period 1987–2001, the prevalence of excess body weight was significantly higher in girls than in boys. Noticeably, a slight increase over a period of 4–5 years was observed in the prevalence of excess body weight in 6–7-year-old children born in 1993–1998 and examined between 1999–2005, which was, however, corrected in the following years. These results, combined with the results of the study presented in this paper, show that over the past 20 years, the prevalence of excess body weight in children aged 6–7 years from this region of Poland (Gdańsk) has remained relatively stable. Both studies used the same Polish criteria based on OLAF percentile charts for diagnosing overweight and obesity.

Regardless of the criteria used to identify excess body weight in children and despite the observed slowdown in the trend of increasing the prevalence of excess body weight, the percentage of overweight and obese children remains high. This is a serious problem and a challenge for public health because, as many studies show, the occurrence of childhood obesity not only increases the likelihood of obesity in adulthood but also its complications [1,2,19,20,21,22,23]. This is especially true for cardiovascular diseases and diabetes but also for cancer, asthma, kidney and digestive system diseases as well as mental disorders. Therefore, it is necessary to implement educational, preventive and intervention measures focused on the problem of excess body weight in children.

When compared to other publications, our work is characterized by a long period of observation of a large population of children from a single city, which has been conducted regularly since 1994. Our results clearly show that in the studied population, the prevalence of overweight and obesity is stable, which may be related to the preventive and interventional measures that were undertaken in this population [24,25].

## 5. Conclusions

The prevalence of excess body weight among 6–7-year-old children from Gdańsk remains at a stable level with no increase or decrease over the last 20 years and is higher in girls.

## Figures and Tables

**Table 1:** Mean age, body weight, height, BMI and percentile BMI in study group (SD- standard deviation).

	*n*	Mean Age ± *SD*	Mean Body Weight ± *SD*	Mean Height ± *SD*	Mean BMI ± *SD*	Mean BMI Percentile ± *SD*
Girls	6109	6.53 ± 0.38	23.08 ± 4.14	121.33 ± 5.53	15.56 ± 1.9	47.97 ± 27.42
Boys	6221	6.54 ± 0.38	23.66 ± 4.24	122.35 ± 5.52	15.72 ± 1.9	45.21 ± 27.03
Total	12330	6.53 ± 0.38	23.37 ± 4.2	121.84 ± 5.55	15.66 ± 1.9	46.58 ± 27.26

**Table 2:** Mean age, body weight, height, BMI and percentile BMI in girls by birth year.

Girls	*n*	Mean Age ± *SD*	Mean Body Weight ± *SD*	Mean Height ± *SD*	Mean BMI ± *SD*	Mean BMI Percentile ± *SD*
2001	351	6.72 ± 0.31	23.68 ± 4.32	122.38 ± 5.42	15.72 ± 1.92	49.15 ± 27.76
2002	933	6.52 ± 0.37	23.26 ± 4.26	121.5 ± 5.48	15.67 ± 1.95	48.83 ± 27.73
2003	858	6.54 ± 0.4	23.22 ± 4.18	121.6 ± 5.88	15.63 ± 1.94	48.3 ± 27.44
2004	912	6.54 ± 0.37	23.19 ± 4.36	121.6 ± 5.56	15.59 ± 2	47.33 ± 27.67
2005	825	6.5 ± 0.37	22.96 ± 3.85	121.05 ± 5.43	15.6 ± 1.81	48.7 ± 27.43
2006	847	6.53 ± 0.38	22.84 ± 3.96	120.81 ± 5.37	15.57 ± 1.8	48.1 ± 27.02
2007	704	6.54 ± 0.37	23.36 ± 4.21	121.64 ± 5.51	15.71 ± 1.96	49.48 ± 27.45
2008	476	6.5 ± 0.38	22.5 ± 3.92	120.82 ± 5.45	15.34 ± 1.8	44.03 ± 26.55
2009	203	6.3 ± 0.32	22.06 ± 3.76	119.8 ± 4.98	15.3 ± 1.79	43.92 ± 26.91

**Table 3:** Mean age, body weight, height, BMI and percentile BMI in boys by birth year.

Boys	*n*	Mean Age ± *SD*	Mean Body Weight ± *SD*	Mean Height ± *SD*	Mean BMI ± *SD*	Mean BMI Percentile ± *SD*
2001	413	6.71 ± 0.32	24.01 ± 4.6	123.17 ± 5.69	15.72 ± 1.95	44.43 ± 26.88
2002	911	6.51 ± 0.38	24.03 ± 4.47	122.35 ± 5.52	15.96 ± 2.07	48.32 ± 28.16
2003	888	6.55 ± 0.38	23.67 ± 4.14	122.59 ± 5.31	15.67 ± 1.86	44.68 ± 27.54
2004	949	6.53 ± 0.38	23.68 ± 4.46	122.23 ± 5.59	15.75 ± 1.9	45.29 ± 27.38
2005	830	6.55 ± 0.37	23.65 ± 3.9	122.35 ± 5.31	15.71 ± 1.7	45.51 ± 24.92
2006	842	6.53 ± 0.38	23.23 ± 3.85	121.94 ± 5.47	15.55 ± 1.71	43.41 ± 25.99
2007	742	6.53 ± 0.38	23.63 ± 4.11	122.52 ± 5.25	15.67 ± 1.94	44.47 ± 26.97
2008	465	6.51 ± 0.39	23.55 ± 4.22	122.24 ± 5.38	15.67 ± 1.93	44.53 ± 26.62
2009	181	6.33 ± 0.32	23.29 ± 3.91	121.4 ± 5.76	15.71 ± 1.91	45.4 ± 26.68
